# Asymptomatic Anticoagulant Rodenticide Exposure in Dogs and Cats—A French and Belgian Rural and Urban Areas Study

**DOI:** 10.3389/ftox.2022.907892

**Published:** 2022-05-11

**Authors:** Tarek Mahjoub, Emilie Krafft, Léa Garnier, Amélie Mignard, Christophe Hugnet, Sébastien Lefebvre, Isabelle Fourel, Etienne Benoit, Virginie Lattard

**Affiliations:** ^1^ USC1233 RS2GP, INRAe, VetAgro Sup, University of Lyon, Lyon, France; ^2^ Biochemistry, University of Manouba, National School of Veterinary Medicine of Sidi Thabet, Ariana, Tunisia; ^3^ Clinique Vétérinaire des Lavandes, La Bégude De Mazenc, France

**Keywords:** anticoagulant rodenticides, pets, dogs, cats, asymptomatic, non-target species, exposure

## Abstract

Anticoagulant rodenticides (ARs) are important tools for controlling rodent pests, but they also pose a health threat to non-target species. ARs are one of the most common causes of pet poisoning. However, exposure of domestic animals to subclinical doses of ARs is poorly documented. To study the random exposure of dogs and cats to ARs, feces from animals showing no clinical signs of rodenticide poisoning were collected from a network of French and Belgian veterinarians. We analyzed fresh feces from 304 dogs and 289 cats by liquid chromatography-tandem mass spectrometry. This study showed a limited prevalence of AR exposure in dogs and cats of 2.6 and 4.5% respectively. In both species, access to the outdoors is a risk factor for ARs exposure. In contrast, the sex of the animals did not affect the ARs exposure status. The observation of the ratio of cis and trans isomers suggested primary exposure in dogs, but also in some cats. While primary exposure in dogs appears to be related to the use of ARs as plant protection products, primary exposure in cats may be malicious, as warfarin, an anticoagulant formerly used as a rodenticide and now used only in humans, was found in 4 of 13 exposed cats. Secondary exposure may also occur in cats.Our study showed reduced exposure in dogs and cats, compared to wildlife, which often has high exposure, especially in areas where rodent control is important.

## Introduction

Anticoagulant rodenticides (ARs) have been used worldwide for pest control since the mid-twentieth century (Fisher et al., 2019). Due to this intensive use, many animal species, whether or not targeted by these treatments, are regularly exposed to these compounds. ARs are classified into two generations: the first generation (FGARs) includes warfarin, diphacinone, coumatetralyl and chlorophacinone; these compounds have been used in the past for rodent control, but, due to increasing resistance mechanisms in the target species, they are now being replaced by the second-generation anticoagulants (SGARs) which include brodifacoum, bromadiolone, difenacoum, difethialone, and flocoumafen. SGARs have higher toxicity than FGARs and are much more persistent in tissues (Sánchez et al., 2012). Both generations of anticoagulants have the same mechanism of action, these pesticides break the vitamin K cycle in the liver by inhibiting the activity of vitamin K epoxide reductase; ARs result in a progressive reduction in the pool of vitamin K required for the activation of coagulation factors II, VII, IX, and X (Suttie, 1985; Furie and Furie, 1988) leading to depletion and risk of bleeding.

The widespread use of these anticoagulant rodenticides also exposes non-target species that share the same treated area. This exposure can be observed in two different ways: either a primary or secondary exposure. Primary exposure results from direct ingestion of the poisoned bait whether it is a target or non-target species. Severe primary poisonings with ARs are widely documented and are among the most common poisoning in dogs (Lahmar et al., 2019; Bertero et al., 2020; Avolio et al., 2021; CNITV, 2022; Webmaster, 2022). In France the uses of ARs are divided into two categories, plant protection products (PPPs) and biocides (European Commission, 2022). PPPs are widely used in the field for the protection of crops against rodents while biocides are used to a limited extent in and around buildings. The mandatory use of bait boxes since 2013 has significantly reduced this primary exposure to biocidal products, but primary exposure to PPP products used in the field is possible for wildlife but also for domestic species that might consume these baits. This exposure remained largely possible until 2020, when such use was banned (Campagnol, 2020). Nevertheless, illegal uses are obviously still possible. The other type of ARs exposure is secondary exposure where the animal poisons itself by eating rodents that have been poisoned or chronically exposed to low doses of anticoagulants. This mode of poisoning has been widely studied in wild predators such as foxes, raptors or mustelids (Hosea, 2000; Sage et al., 2010; Elmeros et al., 2011; Nakayama et al., 2019). Such exposures are perfectly conceivable in domestic carnivores. In wildlife, it appears that animals exposed to ARs *via* poisoned rodents are frequently asymptomatic. Although hepatic residues of these pesticides persist for extremely long periods of time, low hepatic concentrations of ARs do not cause hemorrhagic syndromes but could have adverse effects, especially on the immune system (Vidal et al., 2009).

Veterinary professionals typically see asymptomatic animals with recent witnessed exposure to ARs or animals that are bleeding with suspected ingestion or known ingestion and delayed presentation. In domestic animals, especially in domestic carnivores the importance of asymptomatic exposure is totally unknown. In order to try to better understand this issue we propose to determine the prevalence of asymptomatic anticoagulant exposure in dogs and cats.

## Materials and Methods

### Animal Sampling

**FIGURE 1:**
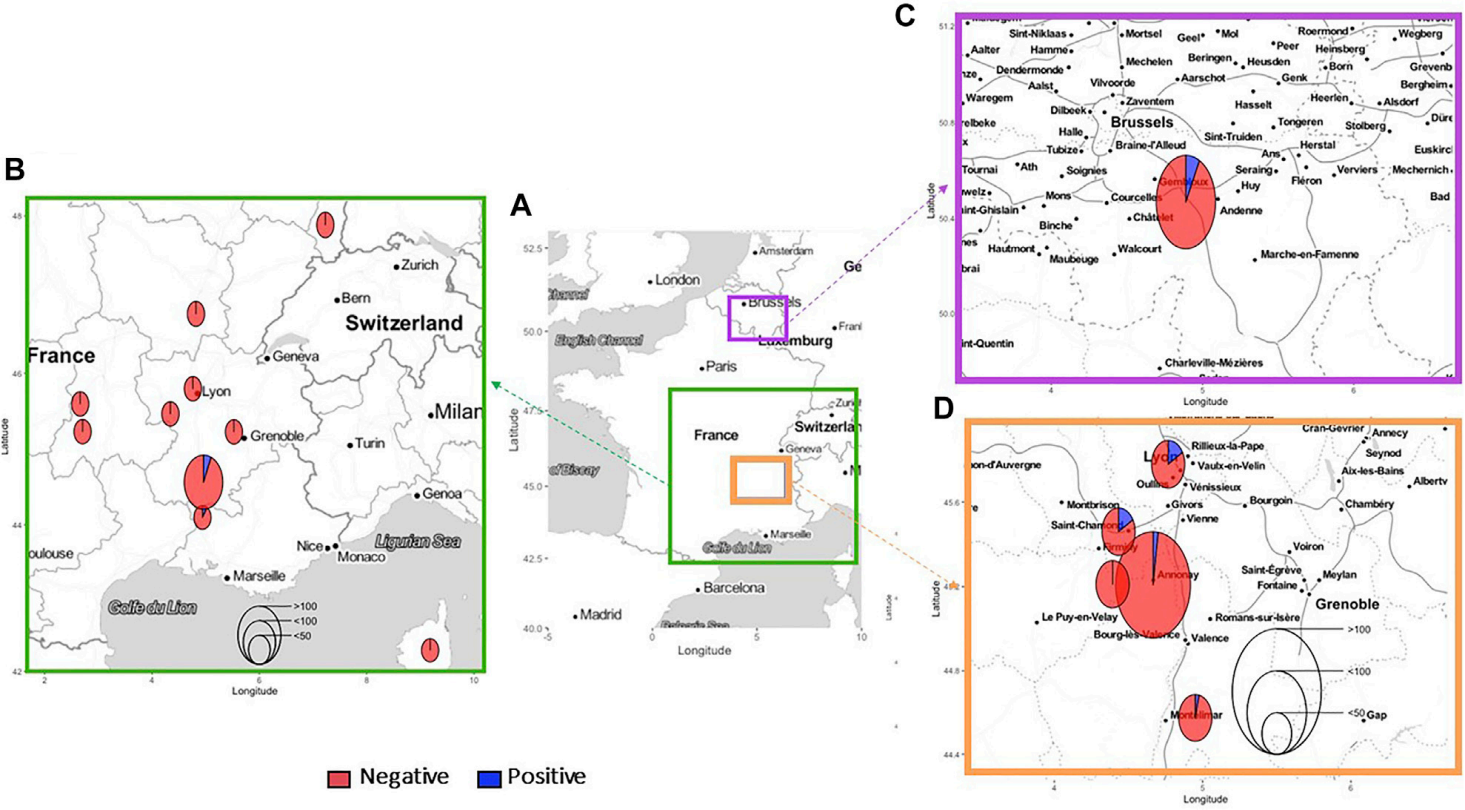
Geographic areas of dog and cat feces samplings. In **(A)** Geographical areas in France and Belgium of dog (in green) and cat (in purple, Belgium; in orange, France) feces collections; In **(B)** zoom on the dog feces collection area. In **(C)** zoom on the cat feces collection area in Belgium. In **(D)** zoom on the cat feces collection area in France. The number of samples per site and the number of positives samples per site are represented by the size of the circle.

To study the random exposure of dogs and cats to ARs, feces from animals showing no clinical signs of rodenticide poisoning (i.e., prostration and/or breathing difficulties and/or difficulty in mobilization and/or traces of bleeding) were collected from a network of veterinarians on animals that were consulted for any other reason not related to a suspicion of exposure to ARs (i.e., sterilization, vaccination, check-up, trauma, infection, metabolic disease, etc). A fresh fecal sample from each individual was placed in a collection pot and accompanied by an information sheet on the animal when this information was known (age, sex, breed, location, reason for visit to rule out any suspect animals, etc). As the sampling of dogs and cats was done at different times and by different people such as students in training and practicing veterinarians, the sampling areas were different (Figure 1).

### Anticoagulant Rodenticides Extraction

A weighing of 0.5 ± 0.05 g of dried feces was performed (i.e., a weight between 0.45 and 0.55 g) and placed in a 50 ml polypropylene tube of Falcon type. 10 ml of acetone was added and vortexed for 30 s. After resting for 1 hour, the tubes were shaken for 10 min by a PTR-60 rotator according to a sequence of cycles with orbital, reciprocal and vortex rotation. Centrifugation at 3000 rpm for 5 min was performed and 6 ml of supernatant was transferred to a test tube. The solvent was evaporated at 40°C under a nitrogen flow. The dry deposit at the bottom of the tube was taken up in 1 ml of acetonitrile, vortexed and added with 1 ml of hexane after 10 min of rest. The supernatant was then removed, and the rest was evaporated at 40°C under a light flow of nitrogen. At that stage, the dry extracts were taken up in 200 µl of methanol. The supernatant was then placed in a vial with a syringe and a 0.2 µm filter. The preparation was then injected (1 µL) in LC-MS/MS for ARs titration according to (Fourel et al., 2018).

### Anticoagulant Rodenticides Analysis

Anticoagulant rodenticides analysis was carried out following the method recently described by (Fourel et al., 2018). The ARs analysed in this study were three FGARs: warfarin, coumatetralyl, chlorophacinone and the five SGARs potentially used in European countries: bromadiolone, difenacoum, brodifacoum, flocoumafen, and difethialone. The chromatographic separation was achieved with a semi-porous Poroshell 120 StableBond C18 column (2.1*100 mm, 2.7 µm) and MS/MS detection was carried out by a 6410 B Triple Quadrupole from Agilent Technologies (Palo Alto, CA, United States) equipped with ElectroSpray Ionization source in negative mode. Two fragment ions were recorded in dynamic Multiple Reaction Monitoring mode and a calibration curve was built up for each analyte. The limits of quantification varied between 1–2 ng/g and the recovery rates were above 70%.

### Data Analysis

Because cats and dogs samplings were done at different periods and in different geographical areas, comparative analysis was not performed. As clinical and epidemiological data were not provided by the sampling veterinarians for all animals, the exposure risk analysis was performed by including only those animals for which epidemiological and clinical data were known. In order to qualify the living environment, three types of zones have been defined: urban zones: corresponding to municipalities with more than 2,000 inhabitants with grouped and collective housing, peri-urban zones: corresponding to municipalities with more than 2,000 inhabitants with individual housing close to an urban center, and rural zones: corresponding to municipalities with less than 2,000 inhabitants.

## Results

### Dog Sampling Description

Three hundred and four fecal samples were collected from dogs between 2016 and 2017 in southeastern France, in 16 different administrative departments, with 1–112 dogs per department (Figure 1, section B). The population descriptors are presented in Table 1. For some dogs, age (*n* = 70), sex and habitat (*n* = 60) and/or department of origin (*n* = 43), were not reported. The breed distribution of the sampled population was very heterogeneous, with a majority of hunting dogs (62.0%) in which 22.7% of the dogs were often used for hunting, followed by companion dogs (24.1%) and shepherds (13.9%). Regarding the geographical distribution of the samples, 58.4% were collected in rural areas, 9.4% in urban areas and 32.2% in peri-urban areas. Regarding the lifestyle of the dogs, the majority (53.2%) lived in a house and had regular access to a garden. A part of the population lived in kennels (28.3%) and 9.8% in apartments. Finally, 8.8% of the individuals lived primarily outdoors on farms.

**TABLE 1:** Description of the dog and cat samples.

	Dog	Cat
Total number	304	289
Date of sampling	2016–2017	2019–2020
Age	(*n* = 238)	(*n* = 166)
Median	5.0	2.0
25–75% percentile	2.0–8.0	1.5–4.0
Min-Max	0.3–16	0.25–19.0
Sex Ratio		
Male	43.4%	16.3%
Female	36.8%	47.4%
Unknown	19.7%	36.3%
Department	(*n* = 261)	(*n* = 289)
Ain	1.5%	−
Allier	8.8%	−
Alpes Maritimes	0.8%	−
Ardèche	5%	63.3%
Corse	0.4%	−
Cantal	6.5%	−
Drôme	43%	9.3%
isère	2.7%	−
Haute Loire	2.3%	2.1%
Haute Loire	−	0.4%
Puy de Dôme	3.8%	−
Haut Rhin	0.4%	−
Rhône	14.6%	2.1%
Saone et Loire	5.7%	−
Vaucluse	4.6%	−
Belgium (Namur)	−	22.8%
Urbanization Level	(*n* = 245)	(*n* = 289)
Rural	58.4%	32.9%
Suburban	32.2%	3.5%
Urban	9.4%	63.7%
Lifestyle	(*n* = 245)	(*n* = 289)
Indoor	9.8%	5.9%
Outdoor	8.2%	65.7%
Indoor/Outdoor	82%	28.4%
AR Exposure	2.6% (i.e., 8/304)	4.5% (i.e., 13/289)
Exposed to 1 compound	2.6% (i.e., 8/304)	4.15% (i.e., 12/289
Multi-exposed	0% (i.e., 0/304)	0.35% (i.e., 1/289)

### Cat Sampling Description

A total of 289 fecal samples were collected in France (77.2%) and Belgium (22.8%) (Figure 1). In Belgium, the cats came from the city of Namur and were all stray cats. In France, the fecal samples came from five administrative departments in the southeast of France, with 1–183 samples per department (Figure 1, section D). The European breed was the dominant breed at 99.3%. One hundred eigthy eight cats were strays, 101 had a known owner. For 105 cats, the sex was not known. For the rest of the sample, the male/female sex ratio was 25.5%/75.5%, respectively. The median age of the population was young (2 years), although for 123 stray cats the age was not known. The age of these strays was estimated by the veterinarian to be between 1 and 3 years. The majority of the cats (63.7%) were from urban areas. 95 were from rural areas (32.9%) and 10 from suburban areas (3.5%). Only 6% of the cats lived strictly indoors.

### Dogs’ Exposure to Anticoagulant Rodenticides

**FIGURE 2:**
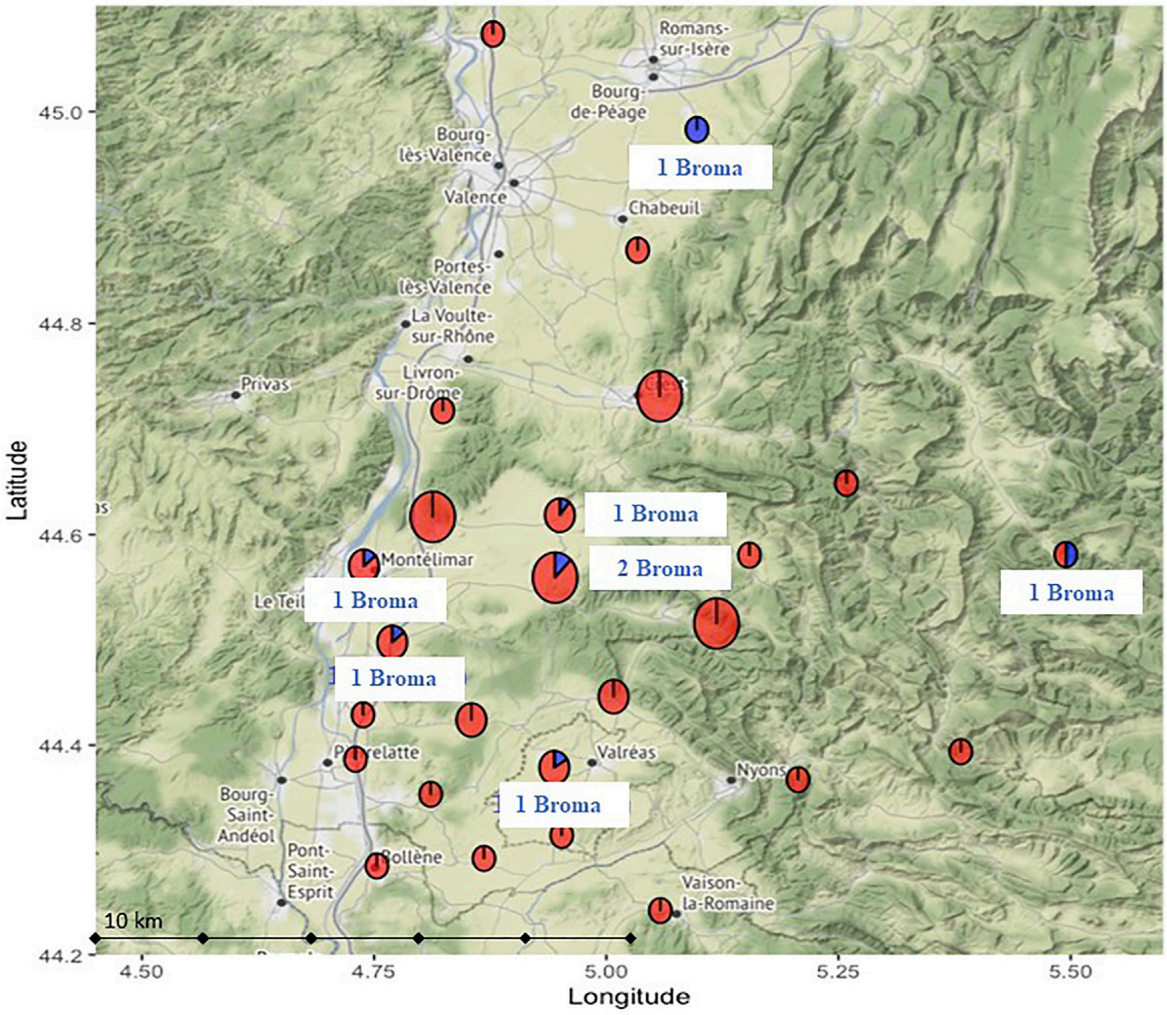
Focus on the dog sample collection area (*n* = 134) near the French town of Montélimar in the Drôme department where the 8 exposed dogs were found, including five in a limited area of only 20 km by 40 km.

Of the 304 dog fecal samples, only 8 contained ARs, a prevalence of 2.6%. Of these 8 samples, only one AR was detected; no multiple exposures were found (Figure 2). Only bromadiolone was detected. All dogs found positive were animals from the same department, Drome, in an area of 100 km by 100 km near the city of Montelimar, including five in an area of only 20 km by 40 km (Figure 1 and Figure 2).

Of the 8 dogs exposed, five were purebred (a Griffon, a Jack Russel Terrier, a Shih Tzu, an English Setter and a Breton Spaniel) and 3 were of mixed breed, 2 from a cross with the beauceron and 1 from the labrador, representing 63% of the hunting dog breed. The average age of the exposed dogs was 7.4 years. Three dogs were females, five were males. All exposed dogs had outdoors access, living in a house or a farm. Five lived in rural areas, two in suburban areas, and one in urban areas. Three dogs were used for hunting.

Median concentration of bromadiolone in feces was 13.41 ng/g (95% CI: 4.64–294.4) with a lower value of 4.64 and upper value of 264.4 ng/g (Figure 4A). Mean percentage of trans-isomers of bromadiolone in feces from exposed dogs was 80.5 ± 10.8% with a lower value of 61% and a higher value of 95% (Figure 4C).

### Cats’ Exposure to Anticoagulant Rodenticides

**FIGURE 3:**
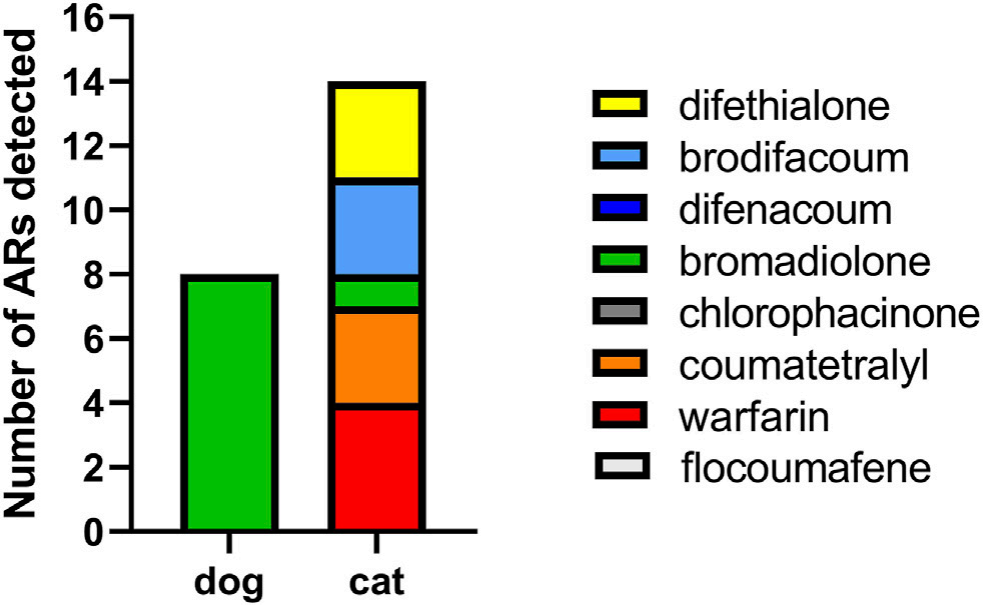
Exposure to AR of cats and dogs. Occurrence of each AR compound in the exposed dogs (*n* = 8) and cats (*n* = 13) with one cat dually exposed to brodifacoum and difethialone.

**FIGURE 4:**
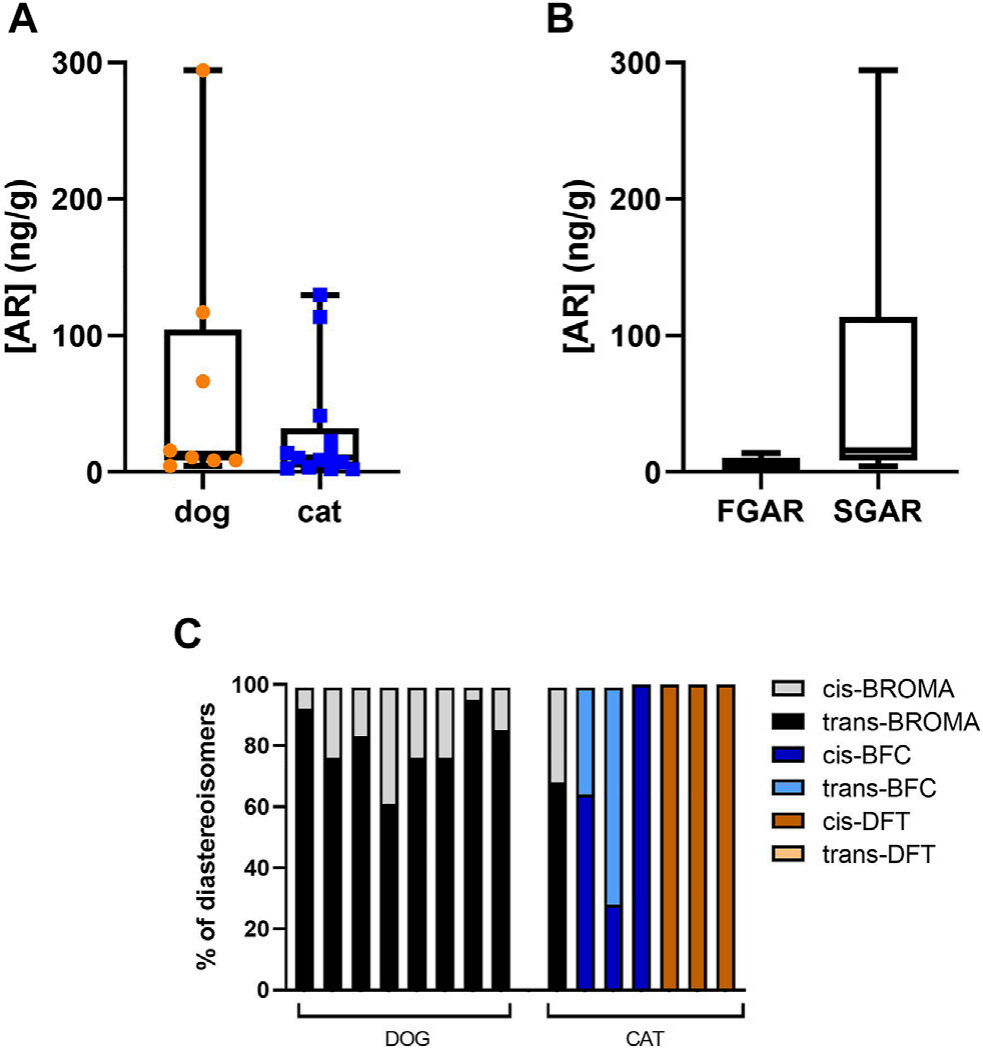
In **(A)** Concentration of AR in feces of exposed dogs and cats. In **(B)** FGARs and SGARs concentration in feces of exposed dogs and cats. In **(C)** *cis* and trans-isomers proportion in feces of each animal exposed to SGARs among the dog and cat samplings.

Of the 289 cat fecal samples, only 13 contained ARs, a prevalence of 4.5%. Of these 13 samples, 12 contained a single AR, one contained two different ARs. Among the samples exposed to a single AR, 7 contained ARs belonging to the FGARs (4 with warfarin and 3 with coumatetralyl) and five contained SGARs (2 with brodifacoum, 2 with difethialone and 1 with bromadiolone) (Figure 3 and Figure 4B). The multi-exposure sample with ARs contained brodifacoum and difethialone.

Eight cats were strays, five had a known owner. All cats had outdoors access, 9 lived exclusively outdoors. Eight cats lived in urban areas, 4 in rural areas, and 1 in suburban areas. Five cats lived in the Ardeche department, including the multi-exposed cat. The compounds detected in these five cats were variable (2 exposures to brodifacoum, 3 exposures to coumatetralyl, 1 exposure to difethialone). Five other exposed cats were stray cats from a parking lot in the city of Namur, Belgium (65 samples from this area). One cat exposed to brodifacoum and 4 cats, collected at the same time, were positive for warfarin. For the 3 remaining cases, two cats, one in the Rhône and one in the Loire, were positive for difethialone and one case was positive for bromadiolone in the Drome department.

Among the 13 AR-positive cats, 4 (2.3%) were neutered (*n* = 119) versus 9 (5.3%) were entire (*n* = 170).

The median concentration of FGARs in feces was 3.6 ng/g with extreme values for coumatetralyl of 2.0 and 13.8 ng/g (Figure 4B). The median SGARs concentration (i.e., brodifacoum, difethialone, and bromadiolone) was 22.6 ng/g with a lower value of 4.1 for difethialone and a higher value of 129.6 ng/g for bromadiolone. The mean percentage of cis-isomers of brodifacoum in the feces of exposed cats was 64 ± 36%, with a lower value of 28% and an upper value of 100% (Figure 4C). The mean percentage of cis-isomers of difethialone in the feces of exposed cats was 100 ± 0% (Figure 4C). The percentage of trans isomers of bromadiolone in the feces of the cat exposed to bromadiolone was 68%.

## Discussion

Asymptomatic exposure of wildlife to anticoagulants has been widely documented. However, exposure of domestic animals has been poorly studied. In addition, severe poisoning cases are very frequently reported in dogs, while very few cases are reported in cats (Walton and Otto, 2018). Besides, the subclinical exposure of these species is poorly documented, with no documented study in cats and only one study in dogs (Seljetun et al., 2020a). In this study, we evaluated asymptomatic exposure of cats and dogs to ARs without preconceived ideas. All samples were taken from apparently healthy animals with no clinical signs of anticoagulant exposure.

Since our objective is to assess the exposure to relatively minimal doses of ARs, the most sensitive biological sample is obviously the liver. However, the sampling is invasive and is not compatible with an exposure assessment study on an live animal. Plasma is very often used, but this biological fluid is not the most appropriate for an ARs exposure study. First of all, blood sampling is still invasive and requires a technical procedure. Overall, its use leads to many false negative results because the plasma circulation of FGARs is very transient and occurs at very low levels, much lower than hepatic concentrations (Vandenbroucke et al., 2008). For example, a recent study shows that the distribution of bromadiolone in plasma at steady state is only 0.4% of the amount present in the body in rats and its presence becomes very quickly undetectable after the initial administration (Chetot et al., 2020). Recent reports have shown that fecal samples, although less sensitive than liver, may be an acceptable and non-invasive alternative for the detection of ARs in animals. Indeed, ARs would be subject to enterohepatic cycling resulting in a long half-life, but also excretion in feces (Watt et al., 2005). This biological matrix has been used to evaluate the pharmacokinetics of bromadiolone in foxes with fecal concentrations 30 to 50 times higher than those of plasma and detectable for more than 25 days post-administration (Sage et al., 2010). A comparative study on the detection of ARs between liver and feces showed that this matrix allowed a satisfactory detection of ARs on a sample of foxes, even if some false negatives are reported (Seljetun et al., 2020b).

In our study, the prevalence of exposure of dogs and cats to ARs were similar and rather low, 2.6 and 4.5%, respectively. This prevalence may be underestimated due to the biological matrix used. Nevertheless, in Norway, 110 healthy dogs were tested for ARs and showed that only one sample contained difenacoum (Seljetun et al., 2020a), whereas the prevalence in non-target wildlife such as foxes was 50% (Seljetun et al., 2019). Indeed, much higher levels of exposure have been reported in wild canids and felids, even when monitoring is performed on feces. Furthermore, a study conducted in France, using fox feces collected in an area where the ground vole is abundant, reported an exposure prevalence of 23% (Fourel et al., 2018). In bobcats in California, exposure has been reported to be close to 39% from plasma monitoring (Serieys et al., 2015). This difference in exposure between domestic and wild canines and felids may be partly due to the inclusion in our sample of animals without access to the outdoors. Nevertheless, after excluding them, the exposure prevalence (3.6% for domestic dogs and 4.8% for domestic cats) are still much lower than those reported for wildlife, considering that 9.8% of the dogs in our population lived in open spaces e.g., farms and 65% were stray cats. This low exposure suggests that the conditions of ARs use, especially the use of a bittering agent in bait (Hansen et al., 1993; Lei et al., 2015) and the use of ARs as a biocide in bait stations since 2013 (Lefebvre et al., 2017), allow to avoid exposure of domestic dogs and cats. Despite these precautions, there are unfortunately cases of poisoning in dogs when baits are improperly stored by their owners or when they are misused.

Some conditions leading to exposure of dogs and cats can be identified from our sampling. All animals exposed to ARs were animals with access to the outdoors. Cases of exposure have been found in both rural and urban areas. Unfortunately, the number of positive cases was not sufficient for statistical analysis. Neither gender nor age seems to be determining factors. Can the exposure be characterized as primary or secondary? Observation of the diastereomeric ratios of SGARs residues may provide an answer. Indeed, each SGARs is a mixture of pair of cis and trans diastereomers with different biological properties (Damin-Pernik et al., 2016; Lattard and Benoit, 2019). In fact, one diastereomer is systematically eliminated more rapidly than the other, at least in rats (Damin-Pernik et al., 2017). Baits contain known and regulated proportions of diastereomers. In Europe, SGARs contain more cis-isomers than trans-isomers, except for bromadiolone. The most abundant diastereomer is consistently the most persistent, so that the residues in the body of a rodent that has consumed a SGARs are composed almost exclusively of the most persistent diastereomer with a change in the proportions of diastereomers relative to the original active substance. An animal consuming a poisoned rodent is then exposed primarily or almost exclusively to the most persistent diastereomer, trans isomer for bromadiolone, cis isomer for all other SGARs. An animal consuming the bait is exposed to the diastereomeric pair, except for difethialone which is composed almost exclusively of cis isomer. The residues found in our SGARs-exposed dogs and cats are composed of both diastereomers, except for one cat exposed to brodifacoum and obviously the cats exposed to difethialone. These results suggest a primary exposure of all 8 dogs and at least 2 of the cats (the one exposed to bromadiolone and the one dually exposed). Nevertheless, the representativeness of the diastereomeric composition of the residues in the feces matrix compared to those in the liver matrix has never been studied. It is possible that the more persistent isomers remain in the liver, which would increase the proportion of less persistent isomers in the feces. Studies will be needed to address this specific point. While primary exposure is easily conceivable in dogs, it is more difficult to imagine in cats. The two cats could have been deliberately poisoned with homemade bait.

For the 8 dogs exposed to bromadiolone, they all came from the same department and from the same geographical area, which was quite small compared to the total sampling area (Figure 2). The samples were all taken between April and July 2017, a fairly short period of time. Outside of this area, no dogs were exposed to AR. It is quite surprising to encounter such a lack of positives outside this zone. Thus, the prevalence in this area reaches locally 6% (8/134 dogs sampled in the area). Bromadiolone could be used at the time of sampling either as a biocide to be used around and in buildings by professionals and amateurs, or as a phytosanitary product for the control of vole populations by farmers (subject to prior prefectural authorization, at low population density, with maximum quantities per hectare not to be exceeded). The use of bromadiolone as a PPP at that time and in that area could not be confirmed, but the exposure results suggest such use with or without authorization.

Concerning the exposed cats, we notice that more than 30% of the positive cats carry coumatetralyl which is a low remanence FGARs and therefore disappears rapidly from the body. This could suggest a regular predatory behavior in these felids. Cats, due to their exploratory instinct, explore a variety of environments which explains the different nature of the identified ARs and the multi-exposure. In addition, neutering status appears to have a slight influence, as entire cats have a positivity of 5.3 versus 3.3% in neutered ones. Sex hormones may influence the lifestyle of felines. It is reported that entire cats are more active, travel longer distances and control a larger territory than neutered cats. In addition, these felids still have an aggressive hunting instinct compared to dogs. Poisoned and less alert rodents can be easy prey (Crowley et al., 2019). It should be noted that in Belgium, some cats are positive for warfarin, an anticoagulant used as a human drug for the prevention of thromboembolic events. This ARs would no longer be used in modern rodent control. The 4 warfarin-positive cats are stray cats that were sampled in a limited urban area in Namur, Belgium. Several hypotheses could explain this observation. It is possible that old stocks of warfarin are still being used as a rat poison, or malicious poisoning or even contamination by wastewater containing residues of warfarin-based drugs consumed by the cats or their prey may be suspected (Gómez-Canela et al., 2014). The source of exposure could be also the consumption of human drugs, as described in cases of childhood poisoning (Brands et al., 2021), this remains unlikely in this case because they were all stray cats. This discovery leads to the questioning of the behavior of these felids that can expose themselves to other medications intended for humans.

Although subtoxic levels of ARs in animals do not cause hemorrhagic syndrome, these chemical residues could be not without consequences for the health of these animals. Reduction in body condition has been reported in raptors and in mustelids at sublethal concentrations (Elmeros et al., 2011; Martínez-Padilla et al., 2017). Some studies in barn owls have shown behavioral aberration after secondary exposure to ARs (Salim et al., 2014). Studies suspect that subtoxic levels of ARs might interfere with the immune response by disrupting leukocyte differentiation (Fraser et al., 2018), besides, other work indicates that ARs increase susceptibility to opportunistic infections in bobcats (Serieys et al., 2018). Nevertheless, experiments have shown that cats exposed to brodifacoum showed transient decreases in the production of certain cytokines, without significant impact on the immune response (Kopanke et al., 2018). Other felids such as lynx were found to be at high risk of exposure, with a record of fetal transfer of several ARs. In contrast, it was noted that there are significant relationships between certain levels of ARs exposure and the incidence of notoedric mange (Serieys et al., 2015). Furthermore, teratogenic effects, and post-partum mortality after exposure to ARs have been reported in pregnant animals (Robinson et al., 2005; Fitzgerald et al., 2018).

## Conclusion

AR are widely used in France and represent the chemical treatment of choice for rodent control. Their use, especially in the fields, inevitably exposes wildlife such as foxes or birds, sometimes leading to poisoning. Concerning our domestic animals, and in particular dogs and cats, anticoagulant rodenticides represent the first cause of poisoning in France (Berny et al., 2010). On the other hand, concerning the environmental exposure of these species, our study shows a limited exposure of dogs and cats, in contrast to wildlife, for which exposure is often high, in regions where rodent control is important. These results suggest a good efficiency of the mitigation measures put in place to protect these species.

## Data Availability

The raw data supporting the conclusions of this article will be made available by the authors, without undue reservation.
